# Cryptic splicing: common pathological mechanisms involved in male infertility and neuronal diseases

**DOI:** 10.1080/15384101.2021.2015672

**Published:** 2021-12-20

**Authors:** Saad Aldalaqan, Caroline Dalgliesh, Sara Luzzi, Chileleko Siachisumo, Louise N Reynard, Ingrid Ehrmann, David J. Elliott

**Affiliations:** Newcastle University Bioscience Institute, Newcastle University, Central Parkway Newcastle, UK

**Keywords:** Cryptic splicing, meiosis, neuronal disease

## Abstract

High levels of transcription and alternative splicing are recognized hallmarks of gene expression in the testis and largely driven by cells in meiosis. Because of this, the male meiosis stage of the cell cycle is often viewed as having a relatively permissive environment for gene expression. In this review, we highlight recent findings that identify the RNA binding protein RBMXL2 as essential for male meiosis. RBMXL2 functions as a “guardian of the transcriptome” that protects against the use of aberrant (or “cryptic”) splice sites that would disrupt gene expression. This newly discovered protective role during meiosis links with a wider field investigating mechanisms of cryptic splicing control that protect neurons from amyotrophic lateral sclerosis and Alzheimer’s disease. We discuss how the mechanism repressing cryptic splicing patterns during meiosis evolved, and why it may be essential for sperm production and male fertility.

Pre-mRNA RNA splicing is a crucial mechanism in eukaryotes and is required to enable expression of protein-coding RNAs (mRNAs) from most mammalian genes. Splicing joins together exons within nascent RNA transcripts, thus creating open reading frames from split genes. Splicing is carried out by a molecular machine called the spliceosome [1]. For accurate pre-mRNA splicing the spliceosome needs to precisely identify short consensus sequences called splice sites at exon-intron junctions and join these together. Because of their short length, sequences similar to splice sites (but not selected by the spliceosome) can occur somewhat frequently within genes. Such infrequently used splice sites have the potential to be selected by the spliceosome but are generally not used, so are referred to as “cryptic” in this review (Figure 1). Cryptic splice site sequences are only weakly recognized by the spliceosome and may be located within repetitive sequences and repressed by nuclear RNA binding proteins [2–4]. However, cryptic splice sites can become activated under certain conditions, including some neurological diseases, and their selection can disrupt production of full-length proteins [5]. Some nuclear RNA binding proteins play key roles in repressing the selection of cryptic splicing patterns within the nervous system. These include TDP43 protein that represses cryptic splicing patterns in neurons but becomes disrupted in amyotrophic lateral sclerosis (ALS) leading to the death of motor neurons [6–9]. Cryptic exons are also included in the hippocampus of patients with Alzheimer’s disease [10]. Through its role in cryptic splicing repression TDP43 has been identified as a “guardian of the transcriptome” that is essential for neuron survival [7]. Whether repression of cryptic splicing is important outside of the nervous system has been less well understood. Here, we highlight recent research that reveal a male germ cell-specific nuclear RNA binding protein that operates as a newly discovered guardian of the transcriptome during meiosis.

**Figure 1.**
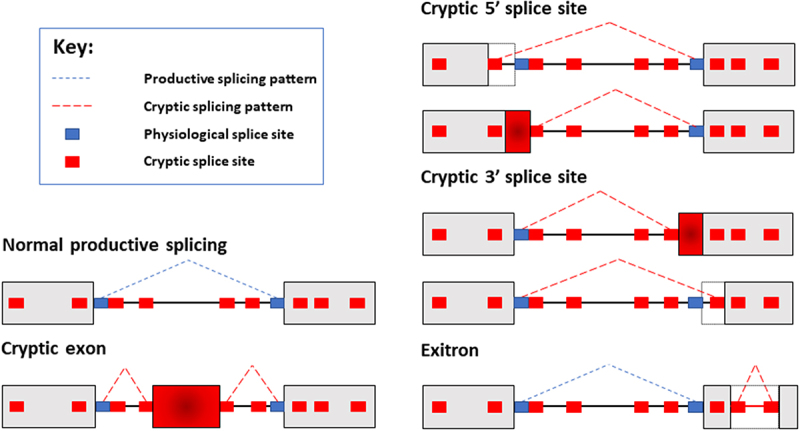
Schematic diagram of cryptic splicing patterns. Most genes are split between exons (shown as gray boxes here) and introns (shown as connecting lines between the boxes). Normal patterns of splice site selection will involve the spliceosome recognizing bona fide splice sites, and joining exons together to create mRNAs. In this example, normal productive splicing is indicated with dashed blue lines. Cryptic splice sites (smaller red boxes) resemble physiological splice sites (smaller blue boxes), and are found within both introns and exons. While normally these cryptic splice sites are ignored by the spliceosome, potentially they could act as decoy sites splice sites for spliceosome selection. Use of cryptic splice sites would produce different mRNAs from genes. Here the normal splicing patterns is shown as a broken blue line joining the physiological splice sites. Examples of cryptic splicing are indicated with dashed red lines. These cryptic splicing events are inclusion of a cryptic exon embedded deep within an intron; selection of cryptic 5ʹ and 3ʹ splice sites; and aberrant recognition of cryptic splice sites within an exon, leading to the interior of this exon being aberrantly recognized as an intron (in a cryptic splicing event known as an exitron).

The testis is considered a relatively permissive site for gene expression patterns. Most human genes produce multiple different mRNAs by using alternative splice sites or by using different combinations of exons. Such alternative splicing permits single genes to produce multiple mRNA isoforms to help amplify the information embedded in the genome. Particularly high levels of alternative splicing have been detected in the testis and in the brain compared to other tissues [11–13]. Alternative splicing patterns can evolve rapidly between species and early analyses detected higher levels of evolutionary divergent splicing in the testis compared to other tissues. This includes the brain, where alternative mRNA isoforms were more likely to be frequently conserved between species than alternative splice isoforms in the testis [11]. More recent comparative transcriptomic analyses confirm some newly evolved exons are exclusively expressed within the testis but suggest there may also be broadly similar levels of conserved mRNA splice isoforms in the testes compared to other tissues [14–16]. The more recently evolved splicing events within the testis are less likely to play a fundamental biological role than more evolutionarily ancient alternative splicing events that have been maintained under selective pressure. However, some recently evolved exons within the testis might later evolve into more generally useful mRNA isoforms via an evolutionary model, which is called the “testis-first” hypothesis. This hypothesis suggests that splicing permissiveness in the testis enables genes to “try out” new exon combinations before they can later be placed under selective pressure [17]. As well as high levels of alternative splicing, there are also particularly high levels of transcription within the testis compared with most other tissues – both in amounts of RNA produced and numbers of genes transcribed [12,18,19].

The human testis produces between 45 and 207 million sperm a day, making it one of the most active developmental pathways still operating in adults [20,21]. The testis contains populations of germ cells (in the developmental pathway leading to sperm) and somatic cells (including Sertoli cells that support germ cell development and Leydig cells that produce testosterone). A population of mitotically active cells called spermatogonia that are early in the germ cell developmental pathway differentiate into cells called spermatocytes. Spermatocytes undergo meiosis, a special form of cell division that produces haploid daughter cells *via* two sequential divisions. The first meiotic division is preceded by a long prophase that lasts around 2 weeks in mice, referred to as meiotic prophase I. This is divided up into five sequential sub-stages called leptotene, zygotene, pachytene, diplotene and diakinesis – all characterized by distinct chromosomal behaviors. During meiotic prophase I chromosomes condense, and non-sister chromatids form crossovers and undergo genetic recombination. Subsequently, cells separate sister chromatids through a second cell division called meiosis II. This produces haploid spermatids that after meiosis differentiate into spermatozoa (Figure 2).

**Figure 2.**
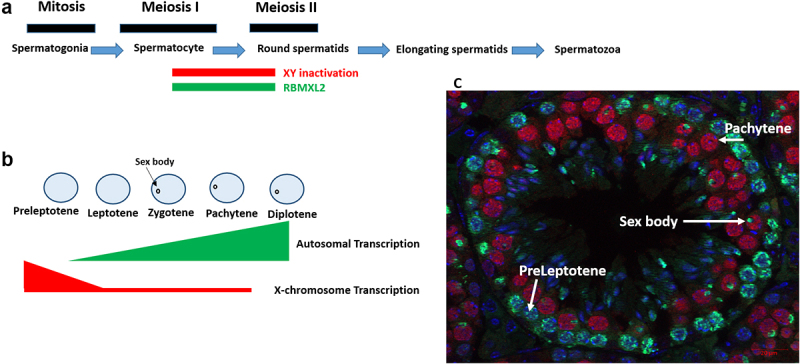
RBMXL2 is expressed during diplotene and pachytene of male meiotic prophase. a. Mouse germ cell development, showing the expression window of RBMXL2 and time period of XY inactivation. b. Transcription patterns of the X chromosome and autosomes during meiotic prophase. c. Seminiferous tubule counterstained with antibodies specific to RBMXL2 protein (pseudocoloured red, detected within nuclei of cells in pachytene in this tubule) and γH2AX (green color, detected within nuclei of pre-leptotene cells and within the sex bodies of pachytene cells).

The cell types that are responsible for the high levels of splicing and gene transcription in the testis have been identified as spermatocytes [12,16,22,23]. Recent transcriptomic analyses of purified testicular cell types reveal that alternative splicing and gene expression levels peak during mid to late pachytene and diplotene stages of meiosis (Figure 2) [24]. In contrast, leptotene, zygotene and early pachytene are transcriptionally quiescent [24–27]. Further RNA sequencing analyses of purified mouse germ cell types detected extensive transcription of both genes and intergenic regions during pachytene and diplotene and in round spermatids, pinpointing these particular cell types as being major contributors to the high levels of testis gene expression and transcriptome complexity [28]. The more “permissive” gene expression environment in the testis may perhaps occur as a result of relaxed chromatin folding. High levels of autosomal transcription during pachytene and diplotene are driven by patterns of open chromatin, including increased levels of the epigenetic mark H3K4me2 (a marker of active promoters) and decreased CpG methylation (a modification normally associated with patterns of gene repression) [12,29]. Furthermore, bursts of meiotic gene expression are driven by activation of super enhancers bound by the MYBL1 and SCML2 transcription factors [30].

A permissive gene environment during meiosis would be consistent with some relaxation of splicing fidelity being tolerated. Despite this, recent data suggest that the interesting parallels between gene expression programs in the brain and testis [13] also extend to a requirement to repress cryptic splicing patterns that would cause cell death. Humans and mice (and likely all placental mammals) express a testis-specific RNA binding protein called RBMXL2 (also known as heterogeneous nuclear ribonucleoproteins G-testis or hnRNP-GT) [31,32]. Mutations have been detected within infertile men for the human *RBMXL2* gene [33]. Mouse RBMXL2 protein is expressed in pachytene and diplotene spermatocytes, the stages of meiosis that have the highest levels of transcription and alternative splicing (Figure 2) [34]. Genetic deletion of the mouse *Rbmxl2* gene causes cell death during meiotic diplotene, thereby reducing testis size and preventing sperm production [34]. Detailed molecular analysis of this mouse model show that RBMXL2 protein prevents the spliceosome selecting cryptic splice sites during the pachytene and diplotene stages of male meiosis [34] – thus performing a similar molecular role to TDP43 in neurons. Both RBMXL2 and TDP43 are members of a group of proteins called hnRNPs (heterogeneous nuclear ribonucleoproteins) that bind to nuclear RNAs as they are transcribed to control their splicing patterns. Although they both operate as “guardians of transcriptomes”, RBMXL2 protein is only expressed in spermatocytes and spermatids. In contrast, TDP43 protein is expressed more ubiquitously. Despite this, point mutations affecting TDP43 specifically cause neuronal cell death [35]. Interestingly, while complete genetic knockout of TDP43 causes embryonic death in mice [36,37], conditional genetic knockout of TDP43 in the testis causes male infertility [38].

Genes encoding mRNAs that are incorrectly spliced in the absence of RBMXL2 are enriched in functions associated with meiosis, chromosome segregation and spermatogenesis (Sara Luzzi unpublished). The inappropriate selection of cryptic splice sites in spermatocytes in the absence of RBMXL2 protein might therefore cause spermatocyte cell death by preventing proper expression of key genes needed for meiosis. RBMXL2 regulates splicing patterns of over a hundred genes during meiotic prophase. Important genes that contain cryptic splice sites that are repressed by RBMXL2 protein include *Brca2* (encoding a DNA repair protein involved in genetic recombination) and *Meioc* (which encodes a cytoplasmic protein that is critical during meiotic prophase) [39–41]. Exactly which RBMXL2 target genes are most important for spermatocyte survival is unknown. Complicating this prediction, the phenotypes caused by splicing errors within a narrow window of meiosis might be different from traditional genetic knockouts that assess when a gene is first needed in a developmental pathway. For example, *Meioc* genetic knockout spermatocytes have an unusually short meiotic prophase and do not reach pachytene or diplotene – the developmental window in which RBMXL2 protein is expressed (Figure 2). Similarly, genetic knockout of the *Brca2* gene prevents germ cell development at an early stage before germ cells enter meiosis [41]. The effects of changing *Meioc* and *Brca2* RNA processing pathways during the narrow window of meiotic prophase when RBMXL2 is normally expressed are not well understood and difficult to predict. An alternative cause of spermatocyte cell death in the absence of RBMXL2 may be through genotoxic damage via formation of RNA:DNA hybrids (R-loops). The loss of splicing factors can cause normally intronic regions to be included within incorrectly spliced mRNAs (rather than being removed by splicing). R-loops form as a result of transcription involving local melting of DNA close to the elongating RNA polymerase, and intronic regions remaining within the pre-mRNA being able to base pair with the melted DNA duplex (forming R loops), leading to DNA damage [42]. In principle it should be possible to potentially correct aberrant splicing patterns caused by loss of RBMXL2 during meiosis. However, while individual cryptic splice sites can be therapeutically targeted using antisense oligonucleotides [43], it would be difficult to use this approach to correct the hundreds of targets that are normally controlled by RBMXL2 during meiosis.

Understanding RBMXL2 function fits into a bigger picture involving the evolution of new genes and regulation of gene expression patterns in the body. The *RBMXL2* gene originated via retro-transposition of an mRNA encoded by the X-linked gene *RBMX* approximately 65 million years ago. As a result of this, the *RBMXL2* gene does not contain introns [32]. RBMX is an RNA binding protein important for controlling splicing, transcription and genome stability [44,45]. Unpaired regions of the X and Y chromosomes (including also *RBMX* gene) become transcriptionally silent during pachytene, within a heterochromatic structure called sex body (or XY body) (Figure 2). This process is termed meiotic sex chromosome inactivation (MSCI) [46], and leads to either reduced or complete loss of sex-linked gene expression for the reminder of meiosis (over ~9 days in mice and even longer in humans [47]). Most retrogenes decay rapidly after they are formed. However, many essential X-linked genes that are transcriptionally silenced by MSCI are functionally replaced by retrogenes that are only expressed in the testis [48].

RBMXL2 is only expressed during and immediately after meiosis, so how are the genes controlled by RBMXL2 in the testis normally spliced in other tissues that do not express RBMXL2? A possible answer is that the splice events that are controlled by RBMXL2 during meiosis might be controlled instead by RBMX in other cell types within the body. In this scenario *RBMXL2* may functionally replace *RBMX* function during meiotic prophase, either as a direct “like for like” replacement or as a more specialized replacement that has evolved to control specific gene expression pathways needed for meiosis [49–51]. However, whether RBMX and RBMXL2 proteins have similar functional activity is not yet fully answered. RBMXL2 mainly operates as a splicing repressor during meiotic prophase in mice. In contrast, global data from human cells has characterized the properties of RBMX protein mainly as a splicing activator that binds to specifically methylated pre-mRNA to slow progression of RNA polymerase II transcription thus facilitating spliceosome function and activating exon inclusion [52]. RBMX is also mutated in the X-linked intellectual disability syndrome Shashi syndrome, where it leads to increased p53 activity and neuronal defects via splicing activation of *MDM4* exon 6 [53].

Why is it important to repress cryptic splice sites during meiosis? Conventional splice sites have evolved to enable exons to be precisely joined together and maintain protein-coding open reading frames. Since cryptic splice sites are usually not used they are not under the same selective pressure as *bona fide* splice sites. Cryptic splice site inclusion into mRNAs often disrupts protein-coding reading frames by introducing premature termination codons (PTCs). In cells that are not dividing by meiosis, PTC-containing mRNAs are degraded by an RNA stability pathway called Nonsense Mediated Decay (NMD) that prevents translation into truncated proteins that could be harmful to the cell. However, uniquely in the testes PTC-containing transcripts can become stabilized. This stabilization occurs during meiosis, thus increasing the likelihood of mRNAs originating from cryptic splicing being translated into potentially toxic proteins. The reason for this stabilization is because of meiotic-associated changes in the NMD pathway. One of the core protein components of the NMD pathway is a protein called UPF3B that is encoded by a gene on the X chromosome that is turned off during meiosis by MCSI. As a consequence its autosomal paralogue gene *UPF3A* becomes active when germ cells enter pachytene [54]. Genetic deletion of *UPF3A* induces meiotic defects in a mouse model showing *UPF3A* expression is critical for meiosis [54]. However, UPF3A protein has only weak activity in the NMD pathway because of an amino acid substitution compared to UPF3B. In fact, while UPF3B promotes mRNA degradation via NMD, UPF3A may operate as an NMD repressor [54]. Hence meiotic expression of UPF3A may lead to translation of some PTC-containing mRNAs and represent a possible reason why it is particularly important to repress cryptic splicing events during meiosis.

Other RNA binding proteins are also essential for splicing control during meiosis and have been recently reviewed [55–57]. However, the molecular defects that appear during mouse meiotic prophase in the absence of RBMXL2 protein, involving a high frequency of aberrantly selected cryptic splicing events, are largely distinct from those that have been identified for other splicing regulators during meiosis. One example is the splicing regulator PTBP2 that is expressed at high levels in spermatocytes [58]. Although PTBP2 protein represses cryptic splice sites in other cell types, its role during germ cell development seems more consistent as a master regulator of developmentally regulated splicing [58,59]. Genetic knockout of *Ptbp2* causes male germ cells to be prematurely sloughed off into the lumen of seminiferous tubules and defects to accumulate in the Sertoli cells cytoskeleton, suggesting impaired interactions between somatic and germ cells without PTBP2 protein [58]. This testicular phenotype correlates with disrupted splicing patterns detected for ~200 genes normally controlled by PTBP2, mainly with roles in Sertoli-germ cell communication. In contrast, more than 60% of the splicing events controlled by RBMXL2 during meiosis involve repression of cryptic splice sites rather than regulation of already known alternative splice events [34].

How does cryptic splicing repression by RBMXL2 integrate with other recently discovered aspects of splicing control during meiosis? High-throughput RNA sequencing analysis of purified meiotic spermatocytes and spermatids show that 10% of the alternative splicing events during meiosis occur via intron retention [23,24,60]. These intron-retained mRNAs play a key role in developmental gene expression. Stable mRNAs containing retained introns are transcribed in spermatocytes and remain nuclear for a few days before being spliced and translated in the post-meiotic stages of spermatogenesis to encode crucial proteins in sperm development [23]. Mechanistically, intron retention involves the repression of splice sites, leading to whole introns being retained within mRNAs. The retained introns detected in spermatocytes and round spermatids have weak splice sites, suggesting a model where the extremely high levels of transcription during meiosis may overload the splicing machinery thus leading to intron retention. Despite RBMXL2’s established role in repressing the selection of cryptic splice sites during meiosis, global changes in intron retention were not detected in the *Rbmxl2* knockout mouse model [34]. However, slower patterns of intron removal during meiosis may make some pre-mRNAs more vulnerable to cryptic exons being mistakenly selected by the spliceosome in mice that do not express RBMXL2 protein.

A key question for the future is why do spermatocytes die without RBMXL2 protein, and to what extent does this resemble neuronal cell death in cells depleted for TDP43 activity? Is cell death caused by the loss of specific important proteins as a result of cryptic splicing events in protein coding mRNAs or does cell death result from genotoxic damage caused by accumulation of R-loops from incorrectly spliced mRNAs (Figure 3)? What is the mechanism by which RBMXL2 represses cryptic splicing patterns? Does RBMXL2 bind to sequences in pre-mRNAs near to cryptic splice sites to sterically occlude the spliceosome? Or does RBMXL2 protein bind and antagonize the function of splicing activator proteins [61] preventing them from activating selection of cryptic splice sites that are otherwise poised for selection by the spliceosome? Could RBMXL2 repress cryptic exons by stabilizing formation of stalled spliceosomes on nascent RNA [62,63] (Figure 3)? Does RBMXL2 only repress cryptic splicing patterns or is it also involved in other aspects of meiosis (Figure 3)? Finally, do RBMX and RBMXL2 perform similar functions in cryptic splicing repression? This last question is of wide importance: RBMX protein has been implicated in controlling chromosome biology and DNA repair as well as splicing and transcription and is also mutated in an X-linked intellectual disability syndrome [44,53,64]. Supporting a wider biological role, RBMX also controls transcription of the *CBX5* gene within leukemia cells [65,66]. Further mechanistic investigations of RBMXL2 and RBMX functions will help to address these issues and should reveal further gene expression pathways that operate during human development and disease.

**Figure 3.**
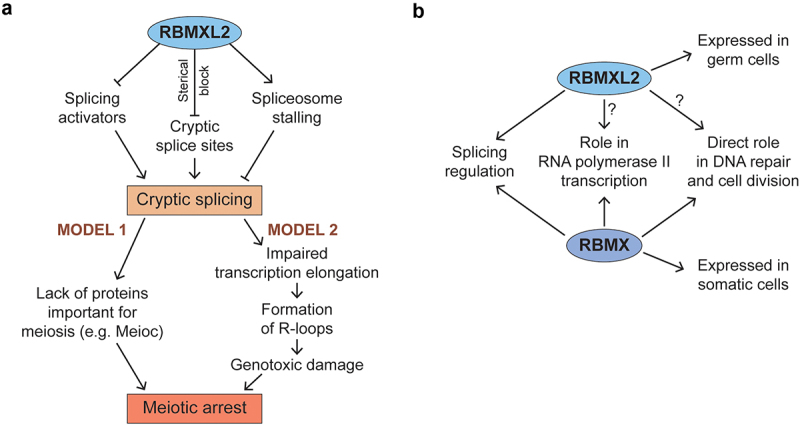
Mechanistic models to explain the impact of cryptic splicing on meiosis, and possible additional roles of RBMXL2. a. RBMXL2 could repress cryptic splicing by either counteracting the function of splicing activators; sterically blocking access of the spliceosome to cryptic splice sites; or promoting stalling of spliceosome assembly at cryptic splice sites. In the absence of RBMXL2 protein cryptic splicing could hinder production of proteins important for meiosis (Model 1) or alternatively impair transcription elongation and promote formation of R-loops (Model 2). Both scenarios would lead to meiotic arrest. b. Both RBMX and RBMXL2 are known to regulate splicing in somatic and germ cells respectively. Could RBMXL2 also have a function in other pathways known to be regulated by RBMX such as RNA polymerase II transcription and DNA repair/cell division?.
